# Photosensitization of A2E triggers telomere dysfunction and accelerates retinal pigment epithelium senescence

**DOI:** 10.1038/s41419-017-0200-7

**Published:** 2018-02-07

**Authors:** Jing Wang, Yiji Feng, Peng Han, Fenghua Wang, Xueting Luo, Jian Liang, Xiangjun Sun, Jing Ye, Yiming Lu, Xiaodong Sun

**Affiliations:** 1 Department of Ophthalmology, Shanghai General Hospital (Shanghai First People’s Hospital), Shanghai Jiao Tong University School of Medicine, No. 100 HaiNing Road, 200080 Shanghai, PR China; 2 International Laboratory in Hematology and Cancer (LIA), Shanghai Jiao Tong University School of Medicine/Ruijin Hospital/CNRS/INSERM/Nice University, Pôle Sino-Français de Recherche en Sciences du Vivant et Génomique, Shanghai Ruijin Hospital, Affiliated to Shanghai Jiaotong University, 200025 Shanghai, PR China; 3 Shanghai Engineering Center For Visual Science And Photomedicine, Shanghai, China; 4 Shanghai Key Laboratory of Ocular Fundus Diseases, Shanghai, China; 5 School of Biology and Agriculture, Shanghai Jiao Tong University, Shanghai, China

## Abstract

Age-related macular degeneration (AMD) is the leading cause of irreversible vision loss in elderly people. AMD is classified as early, intermediate, advanced non-neovascular, and advanced neovascular forms depending on the clinical features. However, the exact pathogenesis remains unclear. Retinal pigment epithelium (RPE) cells degeneration is a hallmark of AMD. With aging, lipofuscin accumulates in RPE cells. N-retinylidene-N-retinylethanolamine (named A2E), a well-known fluorophore of lipofuscin, may contribute to RPE cells degeneration. In this study, we showed that photosensitization of A2E increased DNA damage, including telomere deprotection and deletion, and triggered cellular senescence. In addition, we found that the antioxidant N-acetyl-cysteine (NAC) partially alleviated this DNA damage. Telomerase overexpression rescued A2E-mediated RPE cell senescence, indicating that telomere dysfunction plays an important role in A2E-based senescence. We further showed that the senescence induced by A2E photosensitization may affect the microenvironment of the retina by expressing several factors of the secretory phenotype (SASP) including IL1B, IL13RA2, and CXCR4 through the NF-κB pathway. We propose that expression of these factors create a pro-inflammatory environment that drives retina degeneration. Moreover, our findings suggest that protecting telomeres is a valuable strategy for treating retinal degeneration diseases, such as AMD.

## Introduction

Age-related macular degeneration (AMD) is the leading cause of irreversible vision loss in elderly people in the United States and other developed countries. It is estimated that by 2020, nearly 80 million people will be affected by the disease^1^. The etiology of AMD is believed to result from multiple factors, including sustained oxidative stress, chronic inflammation, as well as predisposing genetic and environmental factors^2,3^. However, the mechanism of AMD remains unclear.

Retinal pigment epithelium (RPE) cells exist as a monolayer between the neural retina and the choroidal vasculature. An important function of RPE cells is to phagocytose the shedding outer segment discs of the photoreceptors to maintain normal visual function. RPE cells also secrete growth factors, such as fibroblast growth factors, transforming growth factor-beta, insulin-like growth factor-I, ciliary neurotrophic factor, vascular endothelial growth factor, and pigment epithelium-derived factor to maintain retina homeostasis^4^. As the retina ages, RPE cells lose their function. A decrease in RPE-mediated phagocytosis leads to an accumulation of lipofuscin, which may potentiate RPE cell degeneration and further promote lipofuscin deposition. RPE dysfunction plays an important role in AMD pathogenesis. Maeda et al. showed that a reduction in the number of photoreceptors following intravitreal ornithine-induced degeneration was directly associated with the loss of RPE cells^5^. In geographic atrophy, Kim et al. showed that the decrease in photoreceptors was associated with a loss of RPE cells^6^.

Age-related lipofuscin accumulation in RPE cells is believed to contribute to AMD pathogenesis^7^. N-retinylidene-N-retinylethanolamine (named A2E) is a well-characterized fluorophore of RPE lipofuscin, which is generated as a byproduct of the visual cycle^8,9^. Sparrow et al. showed that A2E is the major component of lipofuscin that triggers RPE cell damage^10^. Future studies should explore how A2E mediates RPE cell damage in the field of AMD pathogenesis^11^.

Among the aging hallmarks, gradual telomere loss occurring at each cell division acts as a mitotic clock for cellular senescence^12^. For instance, telomeric DNA shortens with each RPE cell division^13,14^. In vertebrates, telomeric DNA consists of repetitive sequences, such as TTAGGG, which form higher order structures such as t-loops and G-quadruplexes^15^. Truncated or dysfunctional telomeres lead to chromosomal damages, transcriptional changes, and cellular senescence^16^. Interestingly, photosensitization of A2E stimulates oxidative DNA damage, such as the formation of 8-oxo-guanines^10,17^. Since telomeric DNA is rich in guanine residues, telomeres may be a target for A2E-mediated oxidation. Thus, we hypothesized that photosensitization of A2E could accelerate RPE senescence through telomere damage.

## Results

### Photosensitization of A2E induces DNA damage and cell senescence in RPE cells

**Fig. 1:**
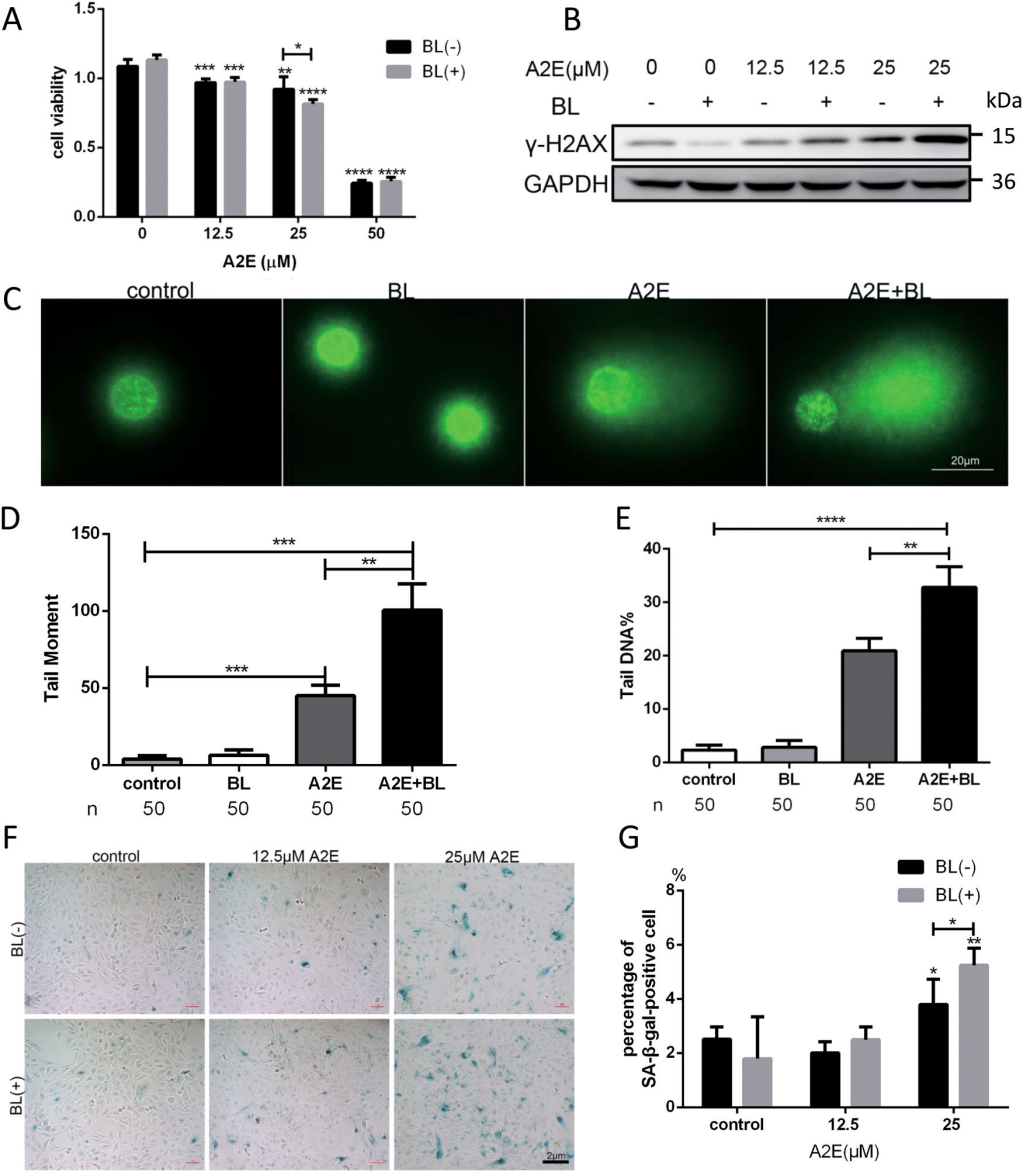
Photosensitization of A2E induced DNA damage and accelerated RPE cell senescence. **a** An MTT assay was performed in RPE cells under specific concentrations of A2E with or without blue light photosensitization. Data are presented as the means ± SD; * indicates *p* value < 0.05, ** indicates *p* value < 0.01, *** indicates *p* value < 0.001, **** indicates *p* value < 0.0001 compared to the control. The experiment was performed at least three times independently. **b** Western blot assay targeting γ-H2AX protein in RPE cells treated with different concentrations of A2E.** c** Representative immunofluorescence microscopic images of the comet assay. Nucleus was stained with YOYO. **d** Statistical analysis of Tail Moment of comet assay shown in **c**; ** indicates *p* value < 0.01, *** indicates *p* value < 0.001. **e** Statistical analysis of tail DNA% of the comet assay shown in **c**; ** indicates *p* value < 0.01, **** indicates *p* value < 0.0001. **f** Representative microscopic images of β-galactosidase staining in RPE cells with various concentrations of A2E. PDL = 10.** g** Quantification of percentage of cells with positive SA-β-gal staining. * indicates *p* value < 0.05, ** indicates *p* value < 0.01

To determine the concentration of A2E that affects the viability of RPE cells, cells were incubated with increasing amounts of A2E for 2 h. Subsequently, the auto-fluorescence of A2E in RPE cells was measured using an immunofluorescence microscope. The results showed that A2E was phagocytized by RPE cells (Fig. SP1). After 24 h in fresh medium, cell viability was examined using a cell viability assay. The viability of RPE cells decreased with increasing concentrations of A2E (Fig. 1a). Interestingly, at 25 μM A2E, blue light photosensitization further decreased cell viability. Interestingly, this A2E concentration is similar to the amount of A2E present in RPE cells harvested from human donor eyes^18^. Thus, we used A2E at a concentration of 25 μM in this study.

We explored whether A2E photosensitization could induce DNA damage based on the level of γ-H2AX. We observed an increase in the level of γ-H2AX in cells treated with increasing concentrations of A2E based on western blotting, and this increase was exacerbated in the presence of blue light (Fig. 1b).

We performed comet assays under alkaline conditions to examine the formation of DNA double-strand breaks (DSBs). DNA was labeled using YOYO dye (YOYO-1 iodide) (Fig. 1c) and DSBs were analyzed according to the tail DNA and tail moment, which determines the migration of the DNA fragment. In agreement with the level of γ-H2AX determined by western blotting, treatment with A2E resulted in a sharp increase in DSBs at the single cell level. At 25 μM A2E, the DSB level was higher in the presence of blue light (Fig. 1 d,e).

Since DNA damage is associated with cell senescence, we monitored the senescence-associated beta-galactosidase (SA-β-gal) staining as a marker for cellular senescence. Our results showed that A2E treatment triggered cellular senescence, an effect that was intensified by blue light (Fig. 1f,g). These findings suggest that photosensitization of A2E accelerated the senescence of RPE cells.

### Antioxidant NAC treatment alleviates DNA damage and cellular senescence stimulated by A2E photosensitization

**Fig. 2:**
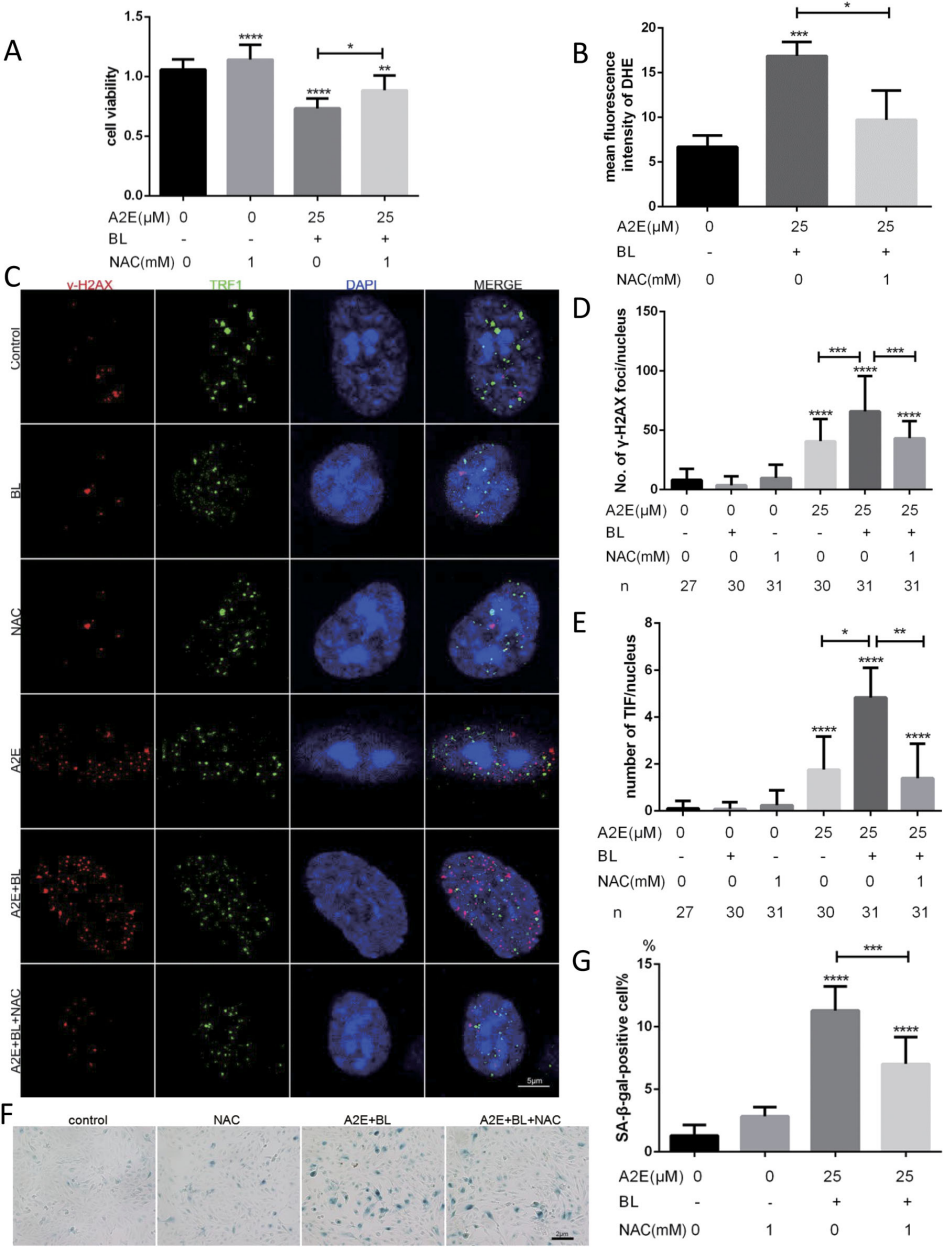
NAC alleviates DNA damage (including telomere deprotection) and partially rescues RPE senescence upon A2E photosensitization by decreasing oxidative stress. **a** An MTT assay was performed in RPE cells treated with 25 μM A2E under photosensitization with or without 1 mM NAC. Data are presented as means ± SD; * indicates *p* value < 0.05, ** indicates *p* value < 0.01, **** indicates *p* value < 0.0001. The experiment was performed independently at least three times. **b** Statistical analysis of the mean fluorescence intensity of DHE by flow cytometry; * indicates *p* < 0.05, *** indicates *p* < 0.001 compared to RPE cells treated with 25 μM A2E under photosensitization. The experiment was performed independently at least three times.** c** Representative images of confocal sections of immunofluorescence assays in RPE cells treated with 1 mM NAC or 25 μM A2E under photosensitization. γ-H2AX foci are labeled red and TRF1 foci are labeled green.** d** Quantification of the number of γ-H2AX foci per nucleus in RPE cells treated with 1 mM NAC or 25 μM A2E under photosensitization. Data are presented as means ± SD; *** indicates *p* value < 0.001, **** indicates *p* < 0.0001.** e** Quantification of TIFs per nucleus in RPE cells with 1 mM NAC or 25 μM A2E under photosensitization. The data are presented as means ± SD; * indicates *p* value < 0.05, ** indicates *p* value < 0.01, **** indicates *p* < 0.0001. **f** Representative microscopic images of β-galactosidase staining in RPE cells treated with 25 μM A2E under photosensitization with or without 1 mM NAC. PDL = 15.** g** Quantification of the percentage of cells with positive SA-β-gal staining shown in **e**; *** indicates *p* < 0.001, **** indicates *p* < 0.0001 compared to control. The experiment was performed independently at least three times

Since the origin of DNA damage could be due to the oxidative stress caused by A2E photosensitization, we treated cells with the antioxidant N-acetyl cysteine (NAC), which is believed to neutralize free radicals before they can harm cells^19^. As expected, we found that NAC alleviated cell growth arrest (Fig. 2a) and decreased the accumulation of reactive oxygen species (ROS) induced by A2E photosensitization (Fig. 2b), as measured by cell viability assays and fluorescence-activated cell sorting (FACS) analysis of dihydroethidium (DHE) fluorescence intensity. Moreover, NAC rescued, at least in part, the pro-aging effects of A2E photosensitization, i.e., γ-H2AX increased (Fig. 2c, d) and SA-β-gal activity (Fig. 2f, g). These results showed that photosensitization of A2E could induce oxidative stress, resulting in DNA damage and cellular senescence.

### Photosensitization of A2E triggers telomere dysfunction

**Fig. 3:**
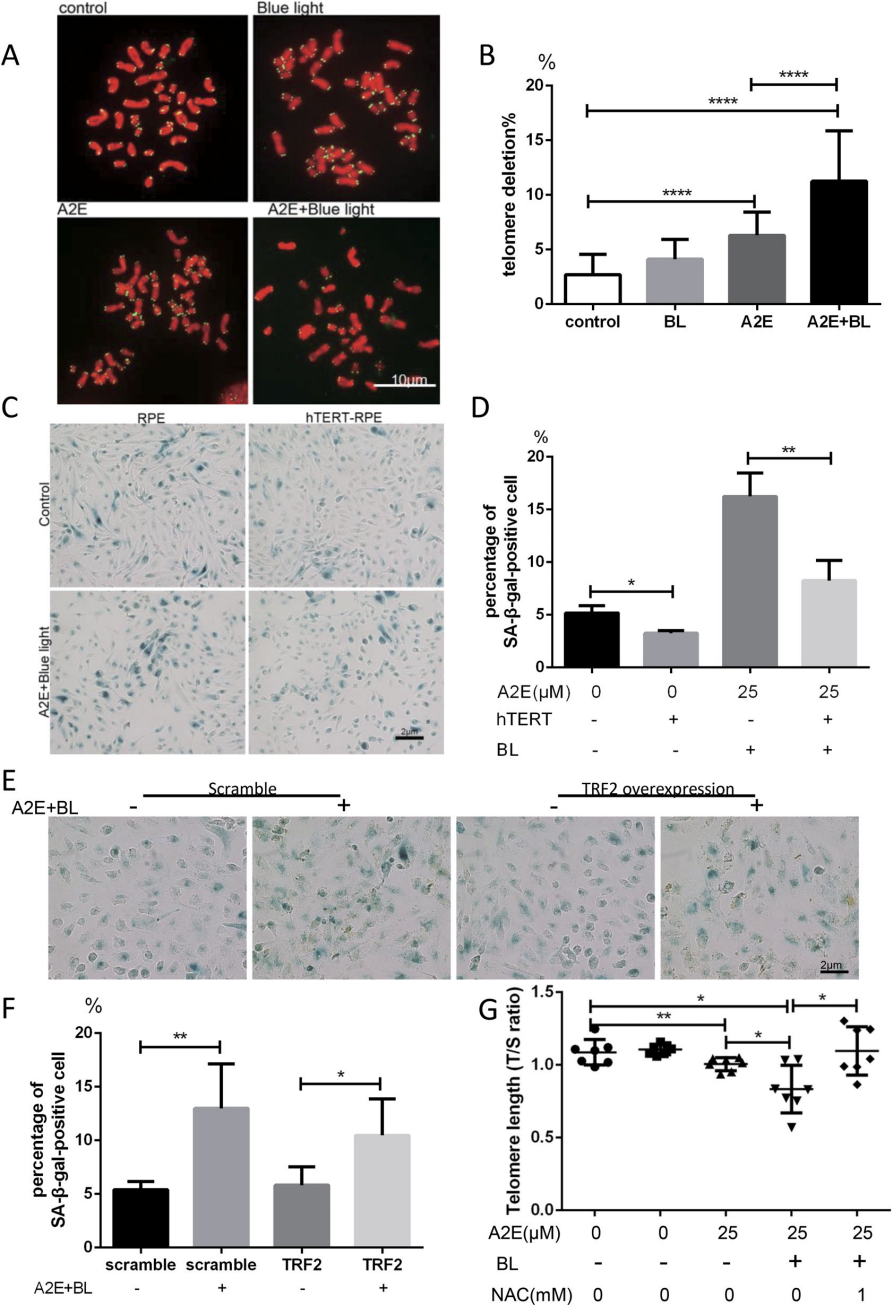
Photosensitization of A2E induced telomere deletions, and ectopic expression of hTERT alleviated cellular senescence in RPE cells, whereas TRF2 overexpression had a minimal effect on RPE senescence. **a** Representative images of telomere FISH in metaphase spreads of RPE cells treated with 25 μM A2E under photosensitization. Telo-foci were stained with a PNA probe and DNA was stained with DAPI. **b** Quantitative analysis of telomere deletion of RPE cells treated with 25 μM A2E under photosensitization. Data are presented as means ± SD. **** indicates *p* < 0.0001.** c** Representative microscopic images of β-galactosidase staining in RPE and hTERT-RPE cells treated with 25 μM A2E under photosensitization. PDL = 32. **d** Quantification of the percentage of cells with positive SA-β-gal staining shown in **c**; * indicates *p* value < 0.05, ** indicates *p* value < 0.01. The experiment was performed independently at least three times. **e** Representative microscopic images of β-galactosidase staining in RPE and TRF2 overexpression-RPE cells treated with 25 μM A2E under photosensitization. **f** Quantification of the percentage of cells with positive SA-β-gal staining shown in **c**; * indicates *p* value < 0.05, ** indicates *p* value < 0.01. The experiment was performed independently at least three times. **g** Telomere length (T/S ratio) measured by RT-qPCR; * indicates *p* value < 0.05, ** indicates *p* value < 0.01 compared to control

Telomeres are particularly sensitive to oxidative stress. Thus, we explored whether telomeres could be a target of A2E photosensitization. First, telomere deprotection was evaluated by monitoring co-localization of the shelterin subunit TRF1 (used as a telomere marker) with γ-H2AX (named TIF for telomere dysfunction-induced foci) (Fig. 2c)^20^. Treatment of RPE cells with A2E increased the number of TIFs per nucleus (Fig. 2e). We examined the telomeres for abnormalities using a telomeric peptide nucleic acid (PNA) probe in metaphase spread staining (Fig. 3a, b). Telomere loss (both single and double strand) was significantly increased upon A2E photosensitization.

Since telomeres were damaged upon A2E photosensitization, we evaluated whether senescence triggered by A2E is the result of telomere dysfunction. Ectopic expression of the catalytic subunit of telomerase (TERT), which may maintain telomeric DNA, reduced the rate of senescence of RPE cells, as monitored by SA-β-gal staining (Fig. 3c, d). Interestingly, TERT overexpression decreased the number of senescent cells in the photosensitized A2E treated group (Fig. 3c, d). However, ectopic expression of TRF2 did not rescue senescence triggered by A2E photosensitization (Fig. 3e, f, Fig. SP2). We measured telomere length (T/S ratio) using RT-qPCR and found that photosensitization of A2E induced significant telomere erosion, which could be decreased by NAC (Fig. 3g). The rate of senescence in TERT-transduced cells remained elevated upon A2E treatment, suggesting that non-telomeric mechanisms were also involved in A2E photosensitization-mediated senescence.

### RPE cell senescence stimulated by photosensitization of A2E triggers the expression of senescence-associated secretory phenotype (SASP) factors

**Fig. 4:**
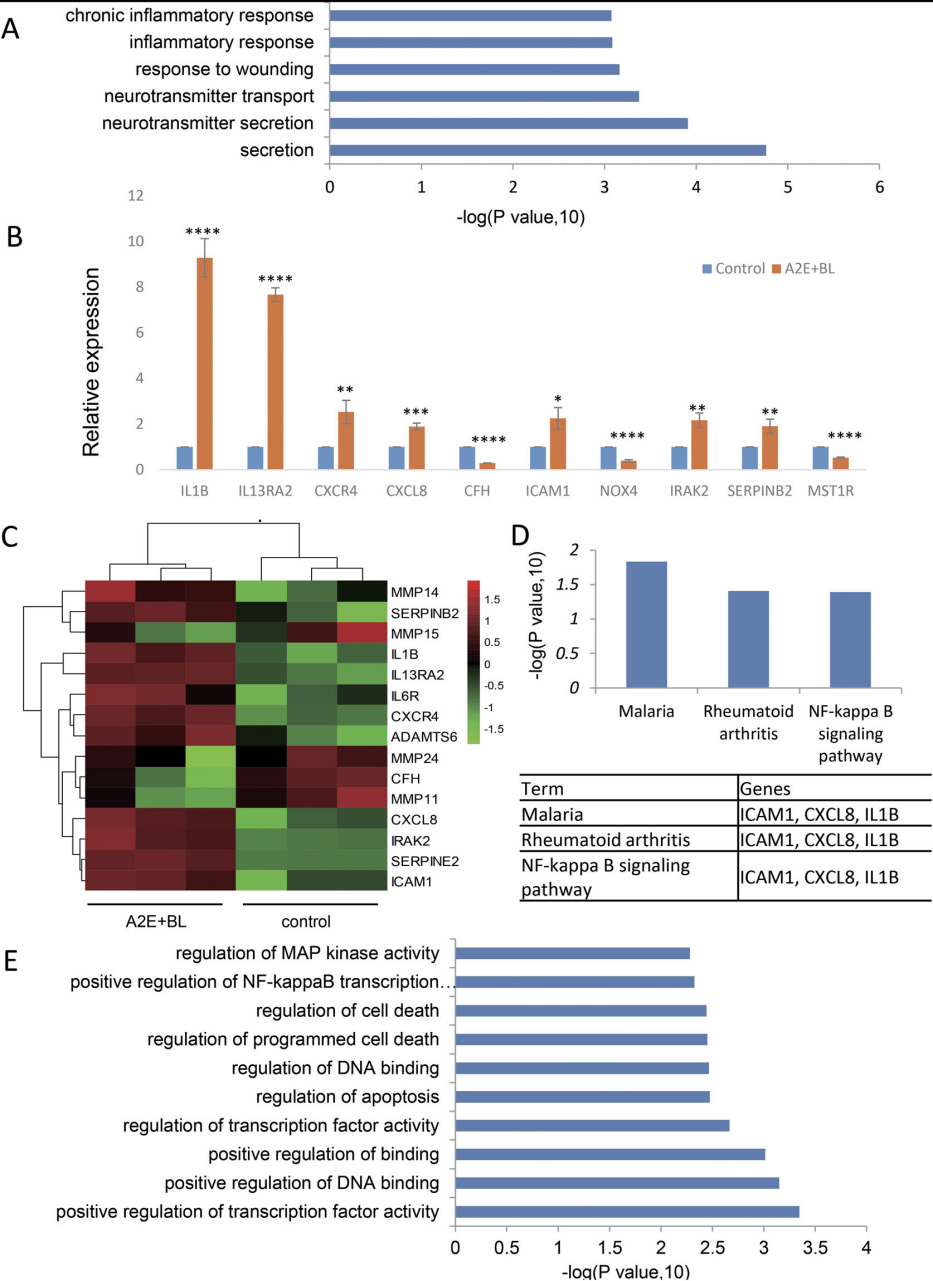
GO analysis and classification of differentially expressed genes. **a** Gene Ontology analysis and classification of differentially expressed genes from control and RPE cells treated with 25 μM A2E under photosensitization into biological processes (top six). **b** Validation of the expression of the pro-inflammatory genes by RT-qPCR; ** indicates *p* value < 0.01, *** indicates *p* value < 0.001, **** indicates *p* value < 0.0001. The experiment was performed independently at least three times. **c** Heat map of genes related to SASP. **d** KEGG pathway analysis of significantly upregulated genes with 25 μM A2E under photosensitization. **e** Gene Ontology analysis and classification of significantly upregulated genes under 25 μM A2E with photosensitization into biological processes (top 10)

To investigate the mechanisms involved in A2E-mediated senescence, we explored how A2E photosensitization influences gene expression using RNA-sequencing (RNA-Seq) and bioinformatics analyses. We considered genes to be differentially expressed if the absolute value of the log2 (fold change) was >1 and the *p*-value <0.05. We identified 130 genes that were differentially expressed upon photosensitization of A2E, including 45 genes that were upregulated and 85 genes that were downregulated. To understand the functions of the differentially expressed genes (DEGs), we performed Gene Ontology (GO) function annotation. The significantly enriched GO terms included three specific categories (biological process, cellular component, and molecular function). We identified the top six enriched GO terms in the biological process (BP) category (Fig. 4a), and the associated genes are presented in Supplementary Table 1. Interestingly, “inflammatory response”, “chronic inflammatory response”, and “response to wounding” were significantly enriched among the BP categories (Fig. 4a)

We selected 10 genes with differing expression profiles for further validation. Their mRNA levels, as measured by RT-qPCR, were consistent with those found in the RNA-Seq analysis (Fig. 4b). The transcript level of IL1β, an important pro-inflammatory cytokine, was markedly increased. These findings are in agreement with a previous study suggesting that IL1β may be associated with AMD^21^. Other inflammatory factors, such as IL13RA2, CXCR4, CXCL8, ICAM-1, IRAK-2, and others, were also significantly increased following A2E photosensitization. Furthermore, we found that other known SASP components, including several matrix metalloproteinases, were downregulated (Fig. 4b, c)^21^. We conclude that A2E photosensitization triggers RPE cell senescence and SASP.

### A2E photosensitization-triggered SASP through activation of the NF-κB pathway

**Fig. 5:**
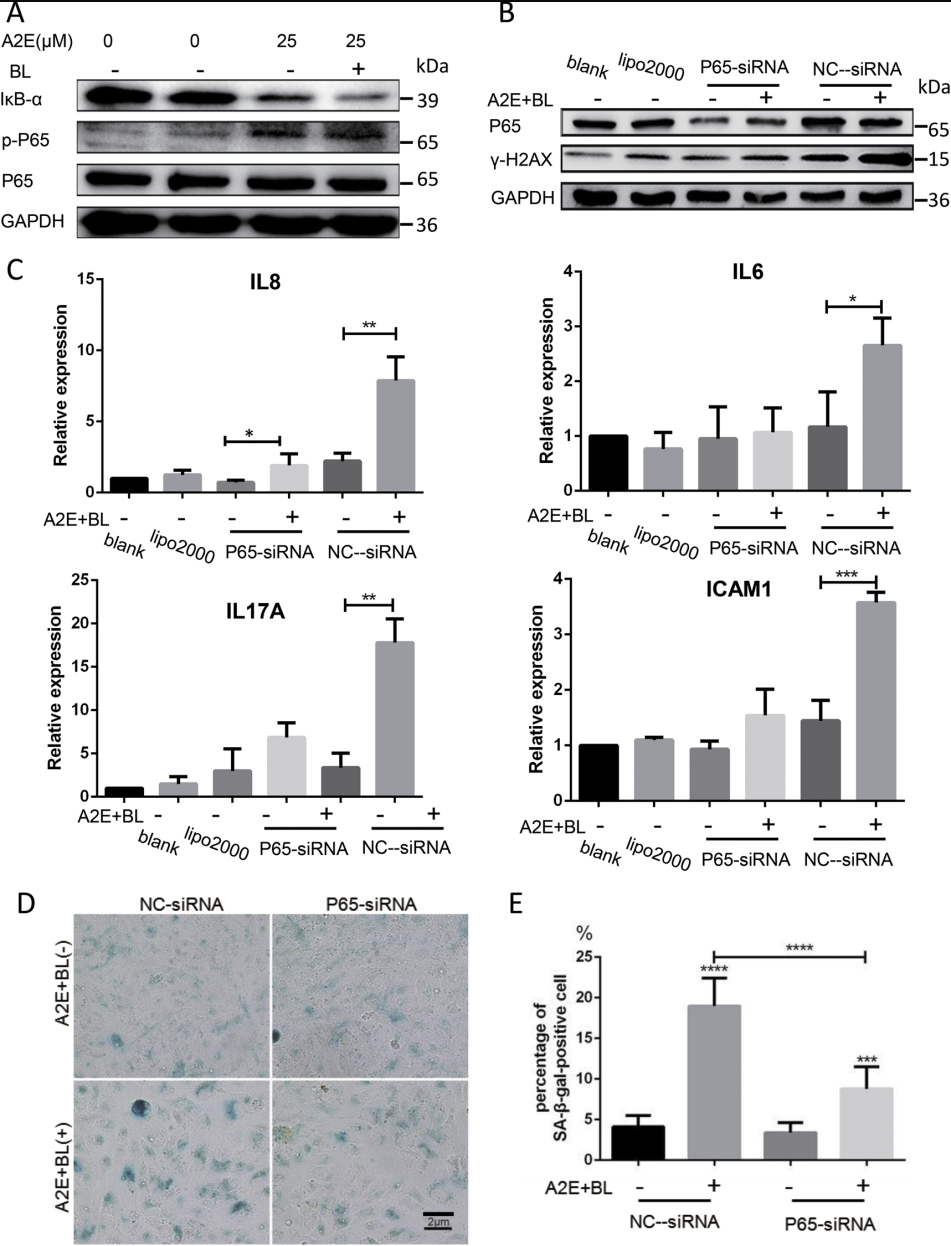
Photosensitization of A2E triggered SASP through NF-κB pathway activation. **a** Western blot assay targeting IκB-α, p-p65, and p65 proteins in RPE cells treated with 25 μM A2E under photosensitization. **b** Western blot assay targeting p65 and γ-H2AX in RPE cells with suppression of p65 functions. **c** Expression of IL8, IL6, IL17A, and ICAM1 in RPE cells with suppression of p65 functions determined by RT-qPCR. Expression is normalized to the expression of β-actin and is expressed as the fold change relative to the control. Values are presented as the means ± SD of two independent samples with triplicate qPCR; * indicates *p* value < 0.05, ** indicates *p* value < 0.01, *** indicates *p* < 0.001. **d** Representative microscopic images of β-galactosidase staining in RPE cells seeded in cell culture carrier plates. RPE cells seeded in cell culture inserts treated with 25 μM A2E under photosensitization with suppression of p65 functions. PDL = 15. **e** Quantification of the percentage of cells with positive SA-β-gal staining shown in **d**; **** indicates *p* < 0.0001 compared to control. The experiment was performed independently at least three times

To increase our understanding of the mechanism governing SASP triggered by A2E photosensitization, we tested for enrichment of KEGG pathway terms. KEGG pathway analysis of the upregulated genes showed a significant enrichment (*p* < 0.05) for three KEGG pathway terms, including “NF-kappa B signaling pathway” (Fig. 4d). GO analysis of the upregulated genes also showed a significant enrichment (*p* < 0.05) for 38 GO terms, including “positive regulation of NF-kappa B transcription factor activity” (Fig. 4e). The top 10 GO terms are presented in Fig. 4e. These findings suggest that the NF-κB pathway is activated upon photosensitization of A2E in RPE cells. Thus, we monitored the total endogenous levels of IκBα, which is phosphorylated at Ser32 and subsequently degraded by proteasomes, resulting in the release and nuclear translocation of active NF-κB. Our results showed that the expression of IκBα was decreased upon photosensitization of A2E. Next, we measured the expression of phospho-p65 (Ser536) and found that it increased upon photosensitization of A2E. However, the total expression of p65 was not affected (Fig. 5a). We further analyzed the nuclear translocation of active NF-κB by immunofluorescence (Fig. SP3). Collectively, these results suggest that the NF-κB pathway was activated.

To explore further whether NF-κB could control SASP stimulated by photosensitization of A2E in RPE cells, we downregulated p65 expression using small interfering RNA (siRNA) in RPE cells (Fig. 5b). We found that knockdown of p65 protein efficiently decreased the expression of key SASP factors, including IL8, IL6, IL17A, and ICAM1 (Fig. 5c). These results are in agreement with previous studies in other models, which showed that NF-κB acts as a master regulator of the SASP^22^.

Recent studies suggest that SASP can reinforce senescence arrest and mediate paracrine effects, triggering senescence in neighboring cells^23^. To explore whether the SASP of A2E-treated RPE cells show this paracrine effect, we used a Transwell system to co-culture A2E-treated senescent cells with untreated A2E cells. After 24 h of co-culture with A2E-treated senescent cells, untreated RPE cells present in the neighboring chamber became senescent based on SA-β-gal activity (Fig. 5d, e). Moreover, the knockdown of p65 in senescent cells caused by A2E photosensitization decreased the rate of senescence induced in the neighboring chamber (Fig. 5d, e). Collectively, these results indicate that paracrine factors produced by senescent A2E-treated RPE cells induced senescence in neighboring cells.

## Discussion

Epidemiological surveys have shown that advanced age is the main risk factor for AMD^24,25^. Aging has been defined as “the progressive accumulation of changes with time that are associated with or responsible for the ever-increasing susceptibility to disease and death which accompanies advancing age”^26^. Cellular senescence can be defined as a permanent arrest of the cell cycle coupled to phenotypic changes, including DNA damage response activation, SA-β-gal overexpression, and the secretion of growth factors, tissue-remodeling enzymes, and various pro-inflammatory molecules forming the SASP^27^. The accumulation of senescent cells in tissues contributes to normal and pathological aging^28^.

Several reports suggest that increased DNA damage and downregulation of DNA repair capacities in RPE cells play a role in the pathogenesis of retina degeneration pathology^29–32^. We confirmed these results by monitoring the level of DNA damage response protein γH2AX in A2E-laden RPE cells. Interestingly, we showed that telomeres are targeted by A2E photosensitization, as revealed by an increased recruitment of DNA damage response factor at telomeres and an increased proportion of telomere loss. In agreement with a causal role of telomere damage with the outcome of A2E-treated RPE cells, the accelerated RPE cellular senescence triggered by A2E photosensitization is rescued by ectopic expression of TERT, indicating that telomere DNA loss plays an important role in photosensitization of A2E-induced RPE cell senescence. The fact that TRF2 overexpression does not rescue the A2E-induced senescence suggests that the telomere alteration triggering senescence does not result from TRF2 dysfunction.

Oxidative stress is believed to play a role in the development of AMD due to the high oxidative stress environment of the fundus^2^. Moreover, telomere length decreases with oxidative stress^33^. Indeed, we found that the antioxidant NAC could partially protect against cellular senescence, as well as the DNA damage caused by photosensitization of A2E in RPE cells. This suggested that A2E-induced cell senescence was a complicated process that partially depends on telomere dysfunction.

Recent studies suggest that senescent cells create a microenvironment through SASP to mediate cross-talk among neighboring cells in a paracrine manner^34^. It has now been shown that SASP components take part in the senescence process. Secreted factors from senescent cells may trigger and/or accelerate senescence process or, otherwise stimulate proliferation and/or transformation of adjacent immortalized cells^35^. IL6, IL8, and other cytokines which attract immune cells play an important role in immune surveilance and subsequent elimination of senescent cells.^36^. According to the above biological process, SASP can facilitate tumor regression or the resolution of wound healing responses^36,37^. Despite the potential importance of SASP in senescence, how the process is regulated in RPE cells remains unclear, and its impact on age-related retina degeneration disease has not been directly assessed.

In this report, we show that photosensitization of A2E changed the gene expression profile in RPE cells based on RNA-Seq analysis. Our results show that the expression of SASP genes was significantly increased. In addition, the pro-inflammatory cytokines IL1β and CXCL8 are associated with AMD pathogenesis^38^. By implicating NF-κB as a major transcriptional modulator in senescence that is required for SASP, our study further supports the importance of SASP in senescence. Indeed, by disrupting p65 (the key mediator of this program), we showed that SASP factors were significantly decreased and that photosensitization of A2E-induced senescence could be rescued. Thus, photosensitization of A2E triggers SASP in RPE cells, which can lead to senescence in neighboring cells. Consequently, our study increases our understanding on the mechanism by which A2E induces RPE cell senescence and the action of NF-κB in age-related retina degeneration disease.

**Fig. 6:**
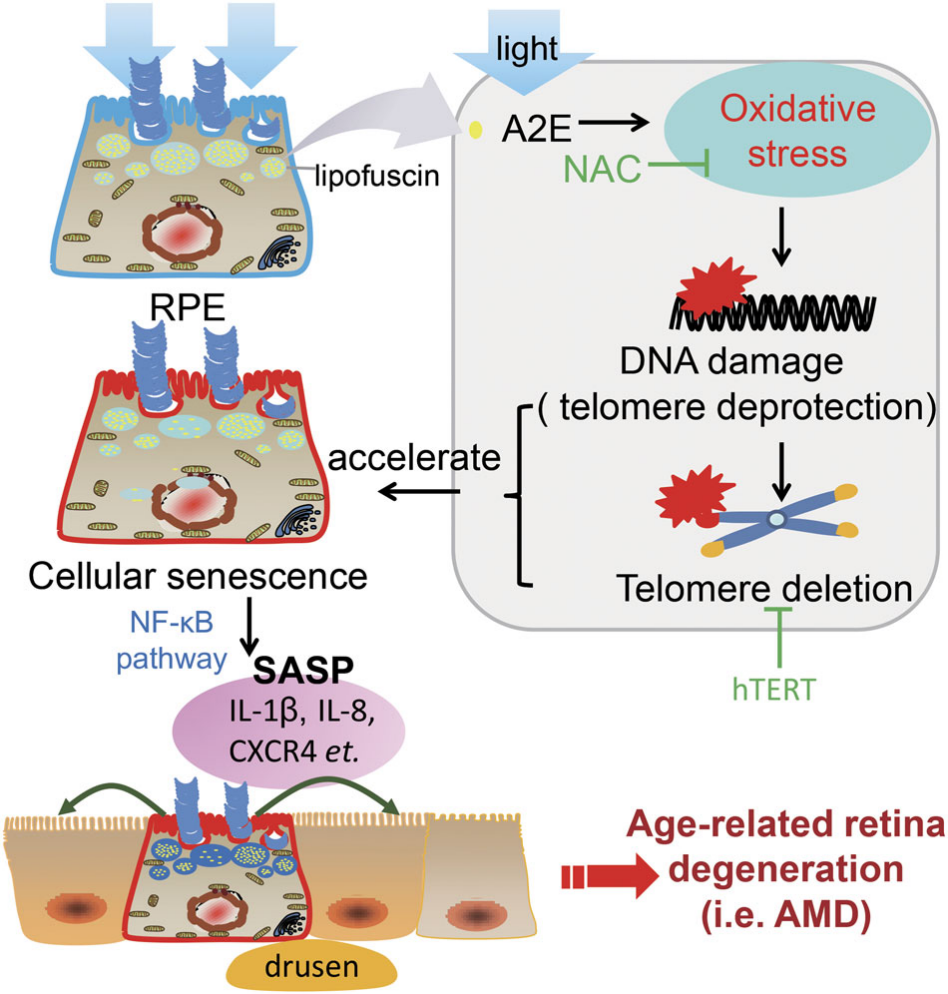
A paradigm of A2E-mediated age-related retina degeneration. In our study, we showed that photosensitization of A2E (which is a byproduct of the visual cycle) accelerates RPE senescence by increasing DNA damage, especially telomere deprotection and deletion. Photosensitization of A2E changed the gene expression profile in RPE cells and significantly increased the expression of SASP genes through the NF-κB pathway. We conclude that photosensitization of A2E triggers SASP gene expression in RPE cells, which may affect the microenvironment of the retina and surrounding cells, contributing to age-related retina degeneration diseases, such as AMD

In conclusion, our study demonstrates that photosensitization of A2E induces DNA damage, including telomere deprotection, further accelerating RPE cell senescence. In addition, we show that A2E photosensitization-induced RPE senescence triggers SASP^39^. The inflammatory environment created by this SASP may trigger and accelerate age-related retina degeneration pathology, similar to AMD. This hypothesis is in agreement with a previous report showing that A2E induced inflammatory chemokine and cytokine production^40,41^. Our results suggest that A2E photosensitization leads to a paracrine effect, affecting the entire retina environment, which may consequently drive retina degeneration pathology (Fig. 6). In addition, our results show that telomere dysfunction is an important cause of A2E photosensitization-induced RPE senescence, suggesting that telomere-protecting molecules may protect against cell senescence^42^.

## Materials and methods

### Cell culture, transfections, and lentivirus production and infection

Human adult RPE cells (APRE-19; American Type Culture Collection, Manassas, VA, USA) were cultured in Dulbecco’s modified Eagle’s/Ham’s F12 (Invitrogen, Grand Island, NY, USA) with 10% fetal bovine serum (Invitrogen), 100 μg/mL streptomycin, and 100 U/mL penicillin at 37 °C under 5% CO_2_ and 95% humidified air. Transfection of plasmid DNA was performed with lipofectamine 2000 reagent (Invitrogen), as described previously. Empty vector or vector expressing TERT or TRF2 were produced by transient transfection of Human Embryonic Kidney 293 Phoenix cell lines with two other packaging plasmids, p8.91 and pVSVg. Next, 48 and 72 h post-transfection, the infectious supernatant was collected and applied to the target cells. The stable hTERT-RPE cell line was selected with 1 μg/mL puromycin for 1 month. The Transwell assay was performed using a NUNC chamber (Cat. No.140640; NUNC, Rochester, NY, USA).

### RNA interference

RPE cells were transfected with double-stranded siRNA or negative control siRNA using Lipofectamine ^TM^ 2000 Transfection Reagent (Cat. No. 116680; Invitrogen, USA). Target sequences were as follows: 5′-GCCCUAUCCCUUUACGUCA-3′ for p65. Double-strand siRNAs were synthesized by Shanghai GenePharma (China).

### A2E formation and treatment

A2E was synthesized from all-trans-retinal (Sigma Aldrich, St. Louis, MO, USA) and ethanolamine at a 2:1 molar ratio, as described previously^43^. A2E was dissolved in dimethyl sulfoxide at a concentration of 25 mM and stored at −80 °C in the dark as a stock solution, as described previously^44^.

RPE cells were incubated with A2E in culture medium for 2 h. Cells were then washed three times to remove extracellular A2E. After A2E loading, RPE cells were exposed to 460 ± 20 nm wavelength light (4000lx; Osram, Augsburg, Germany) for 20 min, as described previously^40^.

### Cell viability assay

The Cell Titer 96 Aqueous One Solution cell proliferation assay (Promega, Madison, WI, USA) was performed as described previously^44^. RPE cells were seeded in 96-well flat-bottomed microliter plates in eight repeat cultures at a concentration of 1 × 10^4^ cells/well. After treatment, each well was incubated with 20 μM MTS assay solution for 2 h at 37 °C and the absorbance was measured using an enzyme-linked immunosorbent assay plate reader at 490 nm emission wavelength. Cell viability was expressed as the percentage of absorbance in cells with indicated treatments to that in cells with solvent control treatment.

### Telomere length assessment by quantitative real-time PCR

Telomere length was measured in genomic DNA extracted from RPE cells by quantitative real-time PCR, as described by Cawthon^45^. The primer sequences were: tel 1, 5′-GGTTTTTGAGGGTGAGGGTGAGGGTGAGGGT-3′; tel 2, 5′-TCCCGACTATCCCTATCCCTATCCCTATCCCTATCCCTA-3′; 36B4u, 5′-CAGCAAGTGGGAAGGTGTAATCC-3′; 36B4d, 5′-CCCATTCTATCATCAACGGTACAA-3′. The RT-PCR reaction volume was as follows: for telomere amplification tell, 30 ng of genomic DNA in H_2_O was added to 10 μL of 2 × SYBR Green buffer, 675 nmol/L Tel-1 primer, 1350 nmol/L Tel-2 primer, and H_2_O to a final volume of 20 μL; and for the amplification of the single copy gene 36B4: 30 ng of genomic DNA in H_2_O was added to 10 μL of 2 × SYBR Green buffer, 800 nmol/L of 36B4u primer, 800 nmol/L 36B4d, and H_2_O to a final volume of 20 μL. The T/S ratio was calculated as follows: [2^CT(telomeres)^/2^CT(single copy gene)^] = 2^−∆CT^. Differences were calculated using Student’s *t*-test at a significance level of 0.05 in Graphpad Prism 6.0 software. All expression analysis was performed for three biological repeats.

### Western blot

Cells were lysed in lysis buffer and boiled for 15 min. Proteins were separated by SDS-polyacrylamide gel electrophoresis, transferred to polyvinylidene difluoride membrane, and probed with antibodies specific for γ-H2AX (1:1000 dilution, #2577, CST), for IκBα (1:1000 dilution, #9242, CST), for Phospho-NF-κB p65 (Ser536) (1:1000 dilution, #3033), p65 (1:1000 dilution, #8242, CST). GAPDH (1:5000, dilution, 60004-1-Ig, Proteintech) antibodies were used to determine the protein amounts as a loading control.

### Immunofluorescence

Slides were either fixed with MeOH at −20 °C or with 4% formaldehyde at room temperature (RT) for 10 to 15 min, and were then incubated with blocking buffer (0.8 × PBS, 50 mM NaCl, 0.5% Triton X 100, 3% milk) for 1 h, followed overnight by incubation at 4 °C with primary antibody to TRF2 (ab217529, 1/100, abcam), to p65 (#8242, 1/200, CST), to γ-H2AX (ab2893, 1/100, abcam) and mouse polyclonal antibody to TRF1 (ab10579, 1/100, abcam) in 0.8 × PBS, 50 mM NaCl, 0.5% Triton X 100, and 3% milk. Cells were then washed three times for 10 min in 0.8 × PBS, 50 mM NaCl, and 1.5% skimmed milk at RT. Incubation with donkey polyclonal anti-mouse ALEXA488 (A21202; Molecular Probes) and donkey polyclonal anti-rabbit ALEXA555 (A31752; Molecular Probes) antibodies was performed for 1 h at 37 °C in the dark in 0.8 × PBS, 50 mM NaCl, 0.5% Triton X 100, and 3% skimmed milk. All antibody incubations were performed in a moist chamber. Cells were then washed three times for 10 min in 0.8 × PBS, 50 mM NaCl, and 0.5% Triton X 100. Slides were then rinsed in PBS, counterstained with 4′,6-diamidino-2-phenylindole (DAPI), mounted in VECTASHIELD, and stored at 4 °C in the dark.

### SA-β-gal staining

The SA-β-gal staining assay was performed using an SA-β-gal staining kit (Beyotime, China) and performed as per the manufacturer’s instructions.

### Comet assay

For each condition, 7000 cells were pelleted and resuspended in 50 μL of 0.5% low melting point agarose dissolved in 1 × PBS at 42 °C. The suspension was immediately laid onto a comet slide (4250-200-03, Trevigen). Agarose was allowed to solidify at 4 °C for 20 min. The comet slides were then immersed in prechilled lysis solution (2.5 NaCl, 100 mM EDTA, 10 mM) at 4 °C for 90 min in the dark. After this treatment, comet slides were placed in a horizontal electrophoresis unit and allowed to equilibrate in electrophoresis buffer for 5 min at 4 °C in the dark. Migration was then performed in 0.5 × TBE buffer (pH = 8) at 40 V for 20 min. Slides were then placed in ethanol (100%) for 30 min at 4 °C (slides can be stored for few days under these conditions). Slides were then thoroughly air-dried and the DNA was stained with YOYO-1 for 10 min.

Comet analysis was performed using the Tritek Comet Score freeware, which measures a wide range of densitometric parameters for each comet. The tail moment (= tail length × DNA in the tail / total DNA) was recorded for each comet (40–50 cells). Student's *t*-test was used to generate statistics.

### Chromosome PNA-FISH

PNA-FISH was performed as described previously^46^. Briefly, cells were washed with PBS and 10 mL of fresh culture medium with 60 μL of colcemid (10 ng/mL) added and incubated for 1 h at 37 °C, after which the cells were collected. Cells were then transferred to a labeled 50-mL tube and centrifuged at 300×*g* for 10 min at 4 °C. The supernatant was then aspirated, leaving 1 mL in the tube, and the pellet was suspended by pipetting. A total of 5 mL of prewarmed KCl (37 °C) was added in a dropwise fashion. Next, 25 mL of KCl was added and mixed by inverting. A total of 100 μL of fresh fixative (methanol/acetic acid = 3/1) was added and mixed. Next, we incubated the tubes at 37 °C for 15 min and centrifuged at 300×*g* for 10 min at 4 °C. The supernatant was then aspirated, leaving 1 mL in the tube, and the pellet was resuspended. We then added 5 mL of fresh fixative in a dropwise fashion and then added an additional 25 mL of fixative and incubated overnight at 4 °C. We next centrifuged the fixed cells at 300×*g* for 10 min at 4 °C and aspirated the fixative, leaving 2 mL in the tube. Next, precooled slides were placed in the humidity chamber and the resuspended cells were added to a slide. We then allowed the slides to dry overnight.

Next, slides were fixed in 4% formaldehyde for 2 min, followed by washing three times. We then prewarmed 50 mL of 0.01 M HCl to 37 °C and added 50 μL/10 mL of pepsin stock (100 μg/μL, Sigma) to it. Slides were then put in the solution and incubated for 10 min at 37 °C, after which the slides were fixed in 4% formaldehyde for 2 min following washing three times for 10 min each. Next, slides were dehydrated by 50%, 75%, and 100% ethanol. Once dry, we applied 120 μL of PNA probe in blocking buffer (70% deionized formamide, 100 mM Tris, pH 7.2, 1% blocking reagent) to a 24 × 60 mm coverslip and touched the slide to the coverslip. The slide was denatured at 80 °C for 3 min, after which the slides were incubated for 2 h at 37 °C. Then, the slide washed twice in washing buffer I (70% deionized formamide, 10 mM Tris, pH 7.2) for 15 min each time. Slides were washed in washing buffer II (0.05% Tween-20, 50 mM Tris, pH 7.4, 150 mM NaCl) three times for 5 min each. We then stained the slides for 3 min in DAPI and washed the slides for 5 min in PBS. Next, VECTASHIELD was applied and covered with coverslips, after which the slides were stored at 4 °C.

### Microscopy

PNA-FISH and comet-FISH assays were recorded on an AxioPlan microscope from ZEISS, equipped with a Plan-Apochrom at 63×, NA 1.4, oil immersion lens, and a cooled CCD camera (CoolSNAP HQ, Photometrics). Image acquisition, processing, and analysis software were from MetaMorph (Molecular Devices). Images of immunofluorescence were recorded using a confocal microscope from Leica.

### ROS labeling and FACS analysis

ROS was labeled using DHE (Invitrogen, Eugene, OR, USA). Cells were incubated with 2.5 μM DHE in the dark at 37 °C for 30 min and then washed three times with PBS and detected by flow cytometry, as reported previously^44^.

### RNA analyses

Total RNA samples were isolated using TRlzol (#15596-018, Invitrogen) and then reverse transcribed into cDNAs using a kit from Takara (#RR047A).

### RNA-Seq and bioinformatics analyses

RNA-Seq was performed according to the manufacturer’s guidelines and previous protocols (C-10365, Life Technologies^47^). RNA deep-sequencing analyses were performed at Epigenetics Key Laboratory of Institutes of Biomedical Sciences (IBS) of Fudan University (Shanghai, China). For bioinformatics analyses, the sequence reads were mapped to human hg19 reference genome transcripts and genome databases using TopHat software allowing 2-bp mismatches per 125-bp seed. Transcript structure and abundance were estimated using Cufflinks software, and differential expression analysis was performed using Cuffdiff software^48^. The cutoff value of differential expression gene was: | log2 (fold change) | > 1, *p*-value <0.05. GO enrichment analysis was performed using DAVID ver. 6.7 (Database for Annotation, Visualization and Integrated Discovery), which is a web-based application (https://david.ncifcrf.gov/)

### Real-time PCR (RT-PCR) validation

RT-PCR primer sequences were designed using Primer3 web software (version 4.0.0). The primer sequences used are provided in Supplementary Table S2. The GAPDH gene was used to calculate the relative fold differences based on comparative cycle threshold (2^−ΔΔCt^) values. The RT-PCR procedure was as follows: 1 μL of cDNA in H_2_O was added to 5 μL of 2 × SYBR Green buffer, 0.1 μM each primer, and H_2_O to a final volume of 10 μL. Differences between the two samples were calculated using Student’s *t*-test at a significance level of 0.05 in Graphpad Prism 6.0 software. All expression analysis was performed for three biological repeats and the average values of three repeats values were shown in the figures.

### Statistical analysis

Based on the univariate test, continuous normal variables were expressed as the mean value ± SD. Parametric variables of normal distribution were analyzed either by the two-tailed *t*-test or the *F*-test of ANOVA, followed by the Duncan test for each two group comparison. Results were considered significant at *p* < 0.05. Statistical analysis was performed with Graphpad Prism 6.0 software.

## Electronic supplementary material

Summary of supplementary data

Supplementary Figure1

Supplementary Figure2

SP. Figure 3

Supplementary Table 1

Supplementary Table 2
