# Supplementary material for: Photosensitization of A2E triggers telomere dysfunction and accelerates retinal pigment epithelium senescence
Source: Cell Death Dis. 2018 Feb 7;9(2):178. doi: 10.1038/s41419-017-0200-7 (PMC5833825; doi:10.1038/s41419-017-0200-7)
Supplement: Supplementary file 1 — Summary of supplementary data [file 41419_2017_200_MOESM1_ESM.docx]

**Summary of supplementary data**

**Supplementary Figure1.** Various concentrations of A2E were phagocytized by RPE cells.

**Supplementary Figure2.** Ectopic expression of TRF2 in RPE cells

**SP. Figure 3** Photosensitization of A2E resulted in P65 translocation into the nucleus.

**Supplementary Table1**.

Shown the differentially expressed genes of the top six GO terms in biological processes.

**Supplementary Table2**.

List of primers sequence.
