# Supplementary figures and images for: Photosensitization of A2E triggers telomere dysfunction and accelerates retinal pigment epithelium senescence
Source: Cell Death Dis. 2018 Feb 7;9(2):178. doi: 10.1038/s41419-017-0200-7 (PMC5833825; doi:10.1038/s41419-017-0200-7)

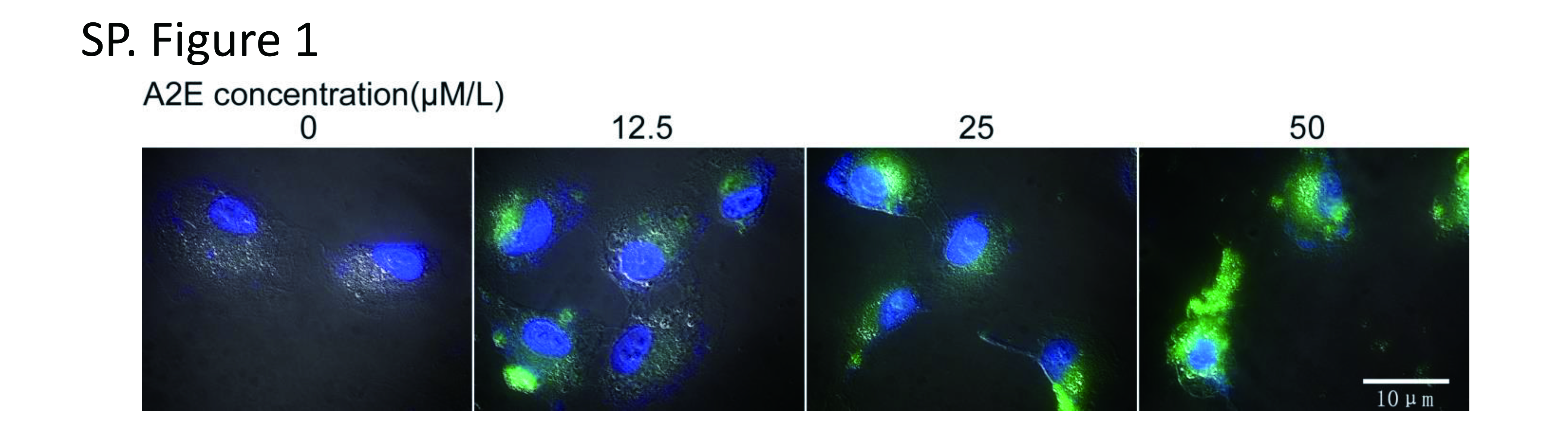

Supplement: Supplementary file 2 — Supplementary Figure1 [file 41419_2017_200_MOESM2_ESM.tif]

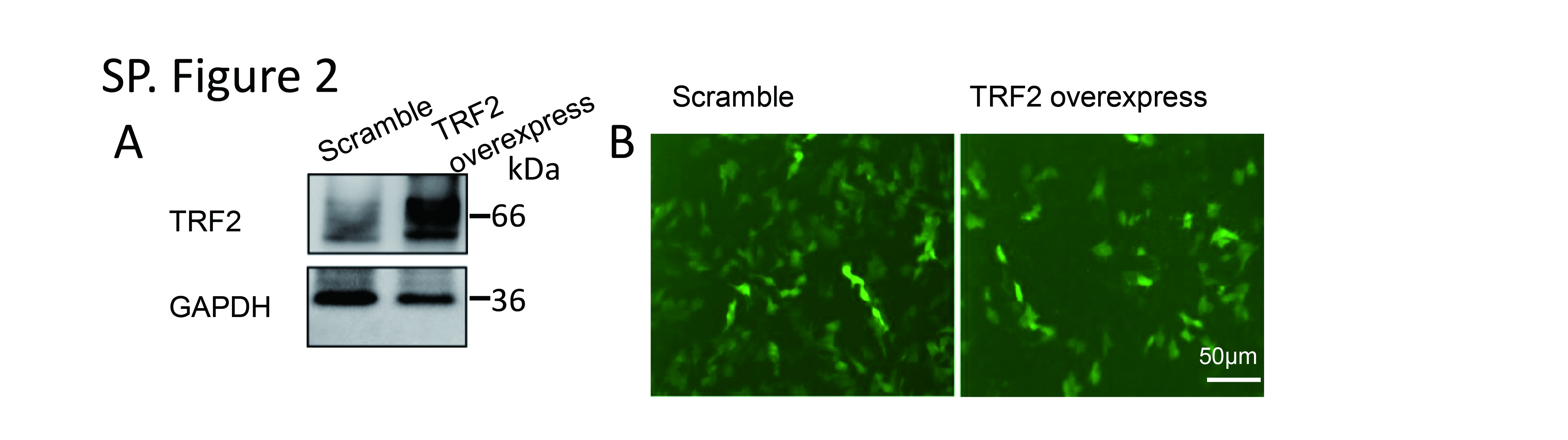

Supplement: Supplementary file 3 — Supplementary Figure2 [file 41419_2017_200_MOESM3_ESM.tif]

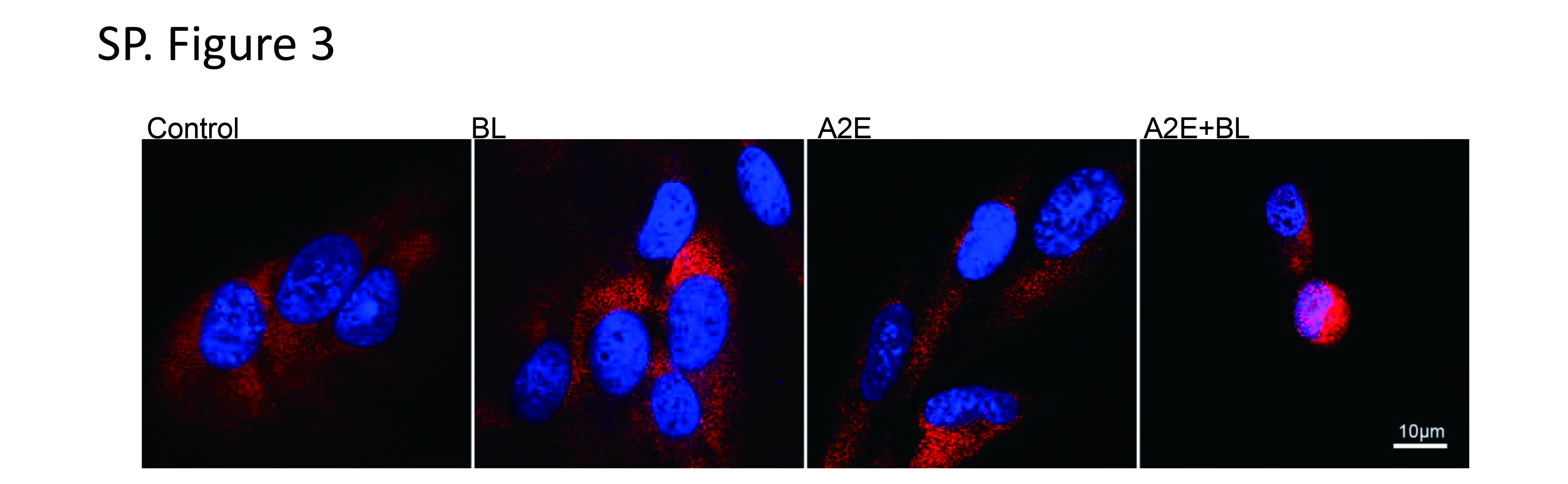

Supplement: Supplementary file 4 — SP. Figure 3 [file 41419_2017_200_MOESM4_ESM.tif]
