# Supplementary material for: Photosensitization of A2E triggers telomere dysfunction and accelerates retinal pigment epithelium senescence
Source: Cell Death Dis. 2018 Feb 7;9(2):178. doi: 10.1038/s41419-017-0200-7 (PMC5833825; doi:10.1038/s41419-017-0200-7)
Supplement: Supplementary file 5 — Supplementary Table 1 [file 41419_2017_200_MOESM5_ESM.doc]

Supplementary Table 1. Shown the differentially expressed genes of the top six GO terms in biological processes

| Term | PValue | Genes |
| --- | --- | --- |
| secretion | 1.73E-05 | SYT1, STX1A, UNC13D, CA9, NRXN3, SYTL5, NPPB, ABCA1, ADORA1, RIMS1, PCLO, SCTR |
| neurotransmitter secretion | 1.23E-04 | SYT1, STX1A, NRXN3, RIMS1, PCLO |
| neurotransmitter transport | 4.21E-04 | SYT1, STX1A, NRXN3, SLC6A17, RIMS1, PCLO |
| response to wounding | 6.87E-04 | IRAK2, NOX4, CYP1A1, ELF3, ADORA1, UNC13D, HMCN1, CXCR4, SERPINB2, SCN9A, CFH, IL1B, THBS1 |
| inflammatory response | 8.27E-04 | NOX4, IRAK2, UNC13D, ELF3, CXCR4, CFH, SCN9A, IL1B, THBS1, ADORA1 |
| chronic inflammatory response | 8.44E-04 | UNC13D, IL1B, THBS1 |
