# Supplementary material for: Photosensitization of A2E triggers telomere dysfunction and accelerates retinal pigment epithelium senescence
Source: Cell Death Dis. 2018 Feb 7;9(2):178. doi: 10.1038/s41419-017-0200-7 (PMC5833825; doi:10.1038/s41419-017-0200-7)
Supplement: Supplementary file 6 — Supplementary Table 2 [file 41419_2017_200_MOESM6_ESM.doc]

Supplementary Table 2. List of primers sequence

| gene | Primer Left | Primer Right |
| --- | --- | --- |
| CFH | tttggaattgatgggcctgc | ttgctcacccgccttataca |
| GAPDH | aggtcggagtcaacggattt | tgacggtgccatggaatttg |
| CXCL8 | gagagtgattgagagtggacc | acttctccacaaccctctgc |
| CXCR4 | ctggccttcatcagtctgga | tcatctgcctcactgacgtt |
| IL13RA2 | agttaaacctttgccgccag | aggtcccaaaggtatgctcc |
| IL1B | tccagctacgaatctccgac | accagcatcttcctcagctt |
| ICAM1 | gtgaccgtgaatgtgctctc | cctgcagtgcccattatgac |
| IRAK2 | tggcctgctacatctaccag | gacttgatcttccgcagctg |
| MST1R | agcttactgagttccaccc | gggcactggtctgagttttg |
| NOX4 | cttcacaactgttcctggcc | gatacttcagcagccctcct |
| SERPINB2 | ggttcatgcagcagatccag | ctgagagagcggaaggatga |
